# Enrichment techniques for clinical metagenomics

**DOI:** 10.3389/fcimb.2026.1723747

**Published:** 2026-05-14

**Authors:** Louis B. David Hanna, Eike Steinig, Katherine Bond, Chuan Kok Lim, Prashanth S. Ramachandran

**Affiliations:** 1 Department of Infectious Diseases at The Peter Doherty Institute, University of Melbourne, Melbourne, VIC, Australia; 2 Victorian Infectious Diseases Reference Laboratory, The Royal Melbourne Hospital at The Peter Doherty Institute for Infection and Immunity, Melbourne, VIC, Australia; 3 Department of Microbiology, Royal Melbourne Hospital, Melbourne, VIC, Australia

**Keywords:** next generation sequencing, metagenomics, enrichment, PCR, hybridisation capture, nanopore adaptive sampling, CRISPR, 2molecular inversion probe

## Abstract

Metagenomic next-generation sequencing (mNGS) offers a powerful, hypothesis-free approach for pathogen detection in clinical samples, allowing the identification of both known and novel microorganisms. However, the predominance of host nucleic acid in most samples poses a significant challenge, often overshadowing low-abundance pathogen sequences and increasing the cost of mNGS due to the high sequencing depth required. Enrichment techniques which selectively amplify pathogen-specific sequences can help to overcome this challenge, improving the sensitivity, specificity, and overall efficiency of mNGS – albeit while compromising the hypothesis-free nature and breadth of shotgun mNGS. As such, they can augment the use of mNGS in clinical scenarios where a more targeted approach is needed. This review provides a comprehensive analysis of the main enrichment techniques currently employed in the field, including PCR-based enrichment, CRISPR-Cas9 enrichment, molecular inversion probes (MIP), nanopore adaptive sequencing (AS), and hybridisation capture-based methods. We evaluate each method on a range of metrics including methodology, cost, sensitivity, specificity, and ease of integration into clinical workflows, as well as describing their application to date for purposes including pathogen detection, antimicrobial resistance profiling, and whole-genome sequencing across diverse clinical sample types. Current limitations and future directions for refinement and implementation of these techniques are also discussed. By summarising the current landscape and latest advancements in mNGS enrichment strategies, this review aims to guide the optimisation of mNGS workflows in clinical diagnostics and highlight key areas for future research.

## 1 Introduction

Over the last decade, the field of metagenomic next generation sequencing (mNGS) for detection of infections from clinical samples has rapidly expanded. As opposed to traditional diagnostic tests which are typically targeted and rely on a prior hypothesis of a possible causative agent, unbiased metagenomic analysis, known as shotgun mNGS, offers the ability to screen for all possible pathogens in a clinical sample, both known and unknown, in a hypothesis-free manner (Miller and Chiu, 2021).

Despite potential, several obstacles currently stand in the way of the widespread application of clinical metagenomics based on shotgun mNGS alone. A major limitation is the heavy predominance of host nucleic acid which can overwhelm the detection of often small amounts of pathogenic DNA, as the high depth of sequencing needed to obtain adequate coverage of such low abundance pathogens can be cost-prohibitive (Walker et al., 2020; Gan et al., 2021; Zakotnik et al., 2022).

Two main strategies to address this are host depletion and pathogen enrichment. Enrichment offers significant advantages when dealing with very low pathogen load samples, albeit at a cost to the fully hypothesis-free diagnostic breadth of shotgun mNGS. As such, the suitability of enrichment for given applications must be evaluated along the spectrum from fully unbiased to fully targeted approaches, accounting for the relevant trade-offs in sensitivity, specificity, limit of detection, and sequencing costs. Enrichment can be performed for various purposes, including pathogen identification, detection of antimicrobial resistance markers, and whole pathogen genome sequencing (Miller and Chiu, 2021).

In this narrative review, we explore the main enrichment techniques currently available to improve pathogen detection, including their methodology, cost, sensitivity and specificity, as well as their performance in the literature to date when applied to a variety of different diseases and clinical sample types. We aim to give a comprehensive overview of the contemporary state of enrichment techniques for metagenomics, providing a useful reference for future studies and helping to clarify gaps in the existing literature.

## 2 Methods

We searched PubMed with the keywords (enrich* OR amplif* OR captur*) AND (next-generation sequencing OR next generation sequencing OR massive parallel sequencing OR massively parallel sequencing) AND (pathogen OR microbe OR virus OR bacteria OR fungi OR parasite). A date range of 01/01/2000 to 01/05/2025 was applied, with results filtered for human studies only. 3446 results were produced, from which 140 were included as potentially relevant after screening. These papers which were included as potentially relevant were those which investigated specific enrichment techniques used to assist the NGS analysis of human infectious diseases from human clinical samples. Non-English and abstract-only papers were excluded, as were papers utilising non-human samples. Further relevant studies from the reference sections of the collected papers and others encountered during the course of the review were also included.

## 3 Results

### 3.1 PCR-based enrichment

PCR-based enrichment uses specific primers designed to amplify sequences of interest in target pathogens. As part of an mNGS workflow, these primers are added to a sample following sample preparation, which then undergoes PCR amplification; the resulting amplified copies of the target sequences, or ‘amplicons’, can then be sequenced using NGS.

PCR-based enrichment for NGS has several benefits. It is a simple, time-efficient technique, taking only a few hours as part of an NGS workflow, and requiring minimal expertise (Brinkmann et al., 2017; Pei et al., 2023). It is also cost-effective, especially for smaller-scale projects, owing to the relative cheapness of reagents, equipment, and probe design (Pei et al., 2023); one study estimated the cost per sample at $78.3USD (Murphy et al., 2023). It also has the ability to sensitively enrich for targets from an extremely low amount of input material and with a low limit of detection; a 256/285-primer panel followed by Ion Torrent Personal Genome Machine (PGM) sequencing could detect targets down to one genome equivalent per sample (Brinkmann et al., 2017).

Nevertheless, several main drawbacks remain. Firstly, very large multiplexed enrichment panels can lead to primer-dimer formation, off-target amplification, and competition for limited reagents (Pei et al., 2023); recent advancements in PCR technology, however, have allowed for increasingly large primer panels. The Paragon Genomics™ CleanPlex Amplicon Sequencing kit can achieve up to 20,000-plex panels, through a clean-up process to remove primer-dimers and off-target amplification products as well as a proprietary panel design algorithm (Pendleton et al., 2019). The short length of PCR primers can also result in a lack of specificity due to being complementary to sequences shared with closely related or off-target organisms (Flaherty et al., 2018; Walker et al., 2020; Fu et al., 2024).

A 13-plex PCR panel targeting AMR-associated regions followed by long-read nanopore sequencing accurately detected drug resistance markers in *Mycobacterium tuberculosis* from clinical respiratory specimens, achieving up to 100% concordance with whole-genome sequencing with only a 2–3 day turnaround (Murphy et al., 2023). Another 19-plex panel targeting AMR-associated loci in *M. tuberculosis*, again followed by long read nanopore sequencing, was able to effectively enrich for *M. tuberculosis* DNA from <0.1% abundance clinical samples, allowing for accurate variant calling (Su et al., 2023).

PCR enrichment followed by NGS has also been applied for whole genome sequencing of several individual pathogens from clinical samples. A multiplex panel followed by Illumina sequencing allowed for the comprehensive characterisation of full-length Hepatitis C virus genomes from plasma samples (Thomson et al., 2016), while a similar protocol allowed for the whole-genome sequencing of tick-borne encephalitis virus from blood and urine samples with low viral loads (Zakotnik et al., 2022). Multiplex PCR enrichment with both short and long read sequencing was also used to sequence whole Zika virus genomes from serum, plasma, and urine samples (Quick et al., 2017), while another study compared the Paragon Genomics CleanPlex SARS-CoV-2 panel with the Illumina *COVIDSeq* Test for SARS-CoV-2 whole-genome sequencing from nasopharyngeal swab samples, with both methods achieving >99% genome coverage (Gerber et al., 2022). A 429-organism PCR panel targeting bacterial, fungal, viral and parasitic respiratory pathogens, followed by Illumina NextSeq 550 sequencing, was applied to bronchoalveolar lavage fluid samples from intensive care unit patients with suspected lower respiratory tract infections for pathogen identification, and compared with traditional methods of pathogen detection involving culture and loop-mediated isothermal amplification assays (Guo et al., 2025). When assessed against patients’ established clinical diagnoses, the targeted PCR-NGS approach achieved a sensitivity of 95.83% compared with 68.75% for the traditional methods. Notably, the targeted method was also able to detect mixed pathogens in 86.00% of patients, compared with 28.00% for the traditional methods (Guo et al., 2025).

For genetic variant analysis, a nested PCR protocol using 2 sets of overlapping primers followed by Illumina MiSeq sequencing was applied to the detection of antiviral resistance markers in Hepatitis B virus from plasma samples (Wang et al., 2017), while a 9-plex reverse transcription-PCR method amplifying the haemagglutinin (HA), neuraminidase (NA) and matrix (M) gene segments, followed by Ion Torrent PGM and MiSeq sequencing, was applied to surveillance of hypervariable regions of influenza A/B genomes (Zhou et al., 2017).

A viral haemorrhagic fever enrichment panel applied to serum samples achieved a limit of detection 10-fold lower (100ge per sample vs >10 ge per sample) than direct NGS for viruses including Yellow Fever Virus, Chikungunya Virus, and Ebola Virus, as well as a 1900-fold increase in the level of Crimean-Congo haemorrhagic fever virus reads (Brinkmann et al., 2017).

### 3.2 CRISPR FLASH

Finding low abundance sequences by hybridisation (FLASH) utilises the CRISPR-Cas9 system to selectively target and amplify sequences of interest within a sample. A set of specifically designed guide RNAs are added to a DNA sample which has undergone blocking through phosphatase treatment, with the guide RNAs directing the Cas9 complex to cleave adjacent to certain target sequences. The newly exposed phosphate ends at these sites can then be ligated with sequencing adapters, allowing subsequent amplification and sequencing using either long or short read NGS.

FLASH-NGS draws on the capabilities of the CRISPR-Cas9 system to be highly flexible and programmable through changing guide RNA design, while also having potential high multiplexing capacity. It can detect and enrich for targets at sub-attomolar concentrations (Quan et al., 2019).

FLASH-NGS achieved 2 to 5-fold enrichment of target sequences in the detection of AMR genes from *Staphylococcus aureus* in respiratory fluid samples as well as from *Plasmodium falciparum* in dried blood spots, while enabling the detection of several AMR genes that were undetectable using regular NGS (Quan et al., 2019). In the enrichment of *Mycobacterium tuberculosis* AMR genes from sputum samples, FLASH-NGS increased DNA recovery and average depth read of target sequences from 1.4% and 6.3 to 33% and 1991, respectively, compared to whole-genome sequencing, whilst demonstrating a sensitivity and specificity of 1.0 in detecting the presence of *M. tuberculosis (*Tram et al., 2023). In a proof-of-concept study applying FLASH combined with real-time nanopore sequencing for the rapid detection of a broader range of AMR genes from respiratory specimens, FLASH demonstrated 2500-fold enrichment of AMR genes compared with regular NGS, while detecting AMR genes that were not detected by regular NGS in 40% of patients’ samples (Serpa et al., 2022). Finally, in a different use of FLASH-NGS, it has been shown to allow for adequate phylogenetic analysis of *Legionella pneumophila* from direct respiratory samples from culture-positive pneumonia patients, as compared to regular whole-genome sequencing from cultured isolates (Domazetovska et al., 2022).

Incomplete coverage of some target sequences represents the main current drawback to FLASH-NGS enrichment; this can potentially be attributed to suboptimal guide design. Thorough and accurate guide RNA design is therefore a crucial consideration in the FLASH-NGS protocol; FLASHiT is a computational tool available for this purpose, which identifies suiTable 20-mer Cas9 Sites and then considers homology between the relevant targeted sequences in order to produce a set of guide RNAs with optimal sequence coverage (Quan et al., 2019). Required DNA input was established as less than 10pg in the original study (Quan et al., 2019). A FLASH-NGS assay utilising real-time nanopore sequencing for the detection of AMR genes from respiratory samples produced results < 6 hours, with the FLASH enrichment protocol adding only 2.5 hours to the total workflow (Serpa et al., 2022). The cost per sample for FLASH has been estimated at $275USD (Tram et al., 2023).

### 3.3 Molecular inversion probes

Molecular inversion probe (MIP) enrichment is a ligation dependent approach utilising probes which hybridise to and circularise around target sequences. Having previously been applied to cancer genomics (Wang et al., 2012) and the characterisation of single-nucleotide polymorphisms (Hardenbol et al., 2005), among other uses, several recent studies have sought to apply MIP enrichment with NGS to direct pathogen enrichment.

MIPs themselves consist of two PCR primer sites, a probe release site for linearisation, and optionally a tag for hybridisation or a ‘unique molecular identifier’, which are flanked by two target-specific sequences. Hybridisation to target regions causes the probe to circularise or ‘invert’. The gap between the two hybridisation sites on the target DNA is then filled via DNA polymerase and joined via DNA ligase. Linear DNA molecules such as non-target DNA and non-hybridised probes are then removed through an exonuclease treatment step, leaving the remaining circular molecules to be amplified either through rolling circle amplification, or linearised and amplified through PCR. The amplicons can then undergo NGS following adapter ligation.

The main benefits of an MIP enrichment approach include the ability for highly multiplexed assays at low cost compared to other techniques, as low as $19USD per sample (Arts et al., 2017), as well as the potential for improved sensitivity and specificity compared with similar methods such as PCR (Hernández-Neuta et al., 2023). The ability of an MIP approach to substantially reduce the costs and effort involved with sample preparation and bioinformatics analysis has also been demonstrated (Hernández-Neuta et al., 2023).

Complexities in appropriate probe design have been implicated in most of the challenges surrounding MIP enrichment. MIP assay sensitivity and specificity are strongly influenced by panel design, with off-target detection between closely related species noted as an occasional problem (Hernández-Neuta et al., 2023). Stringent probe design is therefore crucial (Hernández-Neuta et al., 2023). Other probe design considerations include consistent probe length (typically ~30bp), as the length of the probe backbone affects MIP efficiency, as well as target region GC content, which optimally should be kept consistent (Stefan et al., 2018).

A MIP panel combined with short read NGS achieved 100% sensitivity in detecting targeted bacteria and fungi down to a 1:1000 pathogen to host target ratio, whilst also being highly specific for all but 2 species tested (Hernández-Neuta et al., 2023). For blood culture samples, pathogens and AMRs were accurately detected in 20 out of 24 samples. For 150 CFU/µl spiked *E. coli* blood samples designed to resemble clinical whole-blood samples, the assay was able to detect the pathogen, and importantly there was no evidence of a decrease in read numbers or interference from the blood matrix with increasing amounts of host DNA.

### 3.4 Nanopore adaptive sequencing

Nanopore adaptive sequencing (AS) is a software-based enrichment method using the Oxford Nanopore Technologies (ONT) MinION™ device. First introduced in 2016 (Loose et al., 2016), it utilises the real-time sequencing and analysis capability of nanopore technology to select or reject reads at the level of the sequencing machine, according to their alignment with pre-determined reference criteria. As DNA fragments from the library enter the pore, the first few hundred bases are compared against reference criteria for the enrichment targets. If there is alignment, the entire molecule is further sequenced, whereas if the read is deemed to be off-target, the voltage across the pore is reversed and the DNA fragment is ejected from the pore, allowing that pore to be used to sequence other targets. Therefore, at the level of the resulting sequencing data, there is enrichment for on-target reads and depletion of off-target reads. Importantly, this target enrichment-focused method of AS is distinct from host depletion mode, whereby reads that align to a human reference genome are rejected, while unaligned, non-human reads are sequenced. Such an approach may help to preserve the unbiased breadth of regular metagenomics due to its hypothesis-free nature (Kovaka et al., 2021; Payne et al., 2021).

Several toolkits for the processing and comparison of real-time data against criteria currently exist, including readfish, a base-calling GPU (Payne et al., 2021) and UNCALLED, which enables real-time mapping of raw electrical signals (Kovaka et al., 2021).

To date, AS has shown several advantages as a pathogen enrichment technique. Firstly, as it is software-based enrichment, it can eliminate the need for extensive and complicated sample preparation. Several studies to date have shown that AS can achieve effective enrichment (as high as 14-fold) of targets without any prior PCR amplification of samples (De Meulenaere et al., 2024; Terrazos Miani et al., 2024). Additionally, AS may avoid the problem of amplification bias associated with PCR-based techniques. In the whole-genome sequencing of *P. falciparum*, AS resulted in fewer peaks in sequencing depth across chromosomes, as well as a lower number of chimeric reads, compared to amplicon enrichment (De Meulenaere et al., 2024).

AS can rapidly generate clinically sufficient amounts of sequencing data, in as little as 7 hours (Terrazos Miani et al., 2024). Either by itself or in combination with other enrichment or depletion techniques, AS can produce sufficient sequencing coverage and depth to allow for genome assembly, variant calling, pathogen detection, and identification of antimicrobial resistance markers, and furthermore the enrichment performance of AS has been shown to be superior to that of regular nanopore sequencing (Gan et al., 2021; Lin et al., 2022; Su et al., 2023; De Meulenaere et al., 2024; Terrazos Miani et al., 2024). The current recorded cost of AS has varied from $100USD (Su et al., 2023) and €100 euros (De Meulenaere et al., 2024), up to $500USD (Mordig et al., 2024) per sample tested.

Several challenges currently stand in the way of the wide-scale clinical implementation of AS for pathogen enrichment. Firstly, sensitivity may be an issue in samples with extremely low pathogen load, as the outright enrichment performance of AS is currently lower than other techniques such as PCR enrichment (Su et al., 2023). Furthermore, the enrichment performance of AS is affected when the average fragment length of a library is short (Stefan et al., 2018; Lin et al., 2022; Lin et al., 2023; Su et al., 2023). In these scenarios of libraries with short average DNA molecule length, amplicon-based approaches have been shown to currently have superior enrichment performance (Su et al., 2023). AS can suffer from lower data yield due to the loss of throughput from pores which become distorted due to the rejection of reads (Su et al., 2023) - this can be especially problematic when dealing with samples with very low abundance targets, which can result in the vast majority of pores being lost to rejection-related distortion, limiting the total yield of enriched target sequences (Iyer et al., 2024) As such, enrichment performance is only optimal when target sequences make up between 1-5% of a sample (Iyer et al., 2024). Testing and optimising AS protocols and parameters through real-life runs can also be extremely expensive, due to a cost of up to $500USD per flowcell (Mordig et al., 2024); however, *in silico* simulation tools, most notably Icarust (Munro et al., 2024), can somewhat alleviate this, as can the reuse of flowcells for multiple runs, by stopping runs after sufficient data are obtained and washing the flowcell for subsequent use (Yahya et al., 2023). Finally, consideration needs to be given as to the reference genomes that are used. Integration of proviruses into human reference genomes can result in inappropriate rejection of viral reads (Gan et al., 2021), while the updating of pathogen reference sequences when applying AS to fast evolving outbreaks also needs careful consideration (Terrazos Miani et al., 2024).

Using a combination of enzymatic host depletion, via differential lysis using the HostZERO Microbial DNA kit, and AS, researchers achieved a 113.41-fold enrichment (IQR 23.32 – 327.72) of total microbial DNA when applied to bronchoalveolar lavage fluid samples from children with lower respiratory tract infections, while AS alone achieved a 3.59-fold enrichment (IQR 2.39-10.34) (Gan et al., 2021). When applied to the detection of human adenovirus from nasopharyngeal swab samples, AS combined with rapid PCR-based sample preparation (RPNAS) increased the relative abundance of human adenovirus sequencing data by 7.87 - 12.86-fold (Lin et al., 2022), with this same approach increasing the coverage of SARS-CoV-2 from nasopharyngeal swab samples by 36.68-98.92% compared with standard nanopore sequencing. AS improved detection of *Mycobacterium tuberculosis* from synthetic mock clinical samples compared to regular nanopore sequencing, achieving around 9x coverage as well as up to 3.9-fold enrichment, which was sufficient to allow for variant calling and the detection of antimicrobial resistance markers (Su et al., 2023). A protocol entitled Nanopore Adaptive Sampling with Carrier DNA (NASCarD), which used Lambda bacteriophage DNA as a genomic carrier to prevent the loss of target DNA during library preparation, produced around 1.7x more target reads, a 14-fold increase in the relative abundance and 99% genome completeness in 7 hours of SARS-CoV-2 relevant bases when compared with an equivalent amplicon-based strategy from nasopharyngeal swab samples, which took 72 hours with only 97% completeness (Terrazos Miani et al., 2024). Finally, AS has recently been applied to the whole-genome sequencing of *Plasmodium falciparum* from blood samples (De Meulenaere et al., 2024), achieving enrichment of 2.7 to 5.8-fold from blood samples with levels of parasitaemia between 0.1-0.6%, median sequencing depth ranging from 5-355, and genome coverage as high as 99.9%.

### 3.5 Hybridisation capture-based enrichment

Hybridisation capture-based (HCB) techniques are one of the most widely studied techniques to date to enrich for pathogens from clinical samples for mNGS. The two main types of hybridisation capture are solid-phase capture, where targets hybridise to DNA or RNA probes attached to a solid surface, and solution-based capture, where targets are mixed with biotinylated oligonucleotide probes, or ‘baits’, in solution and allowed to hybridise, before being drawn out via streptavidin-coated magnetic beads. First developed in 2009 (Gnirke et al., 2009), it is the latter method which has been utilised for virtually all studies applying hybridisation capture-based enrichment to human pathogens (Gaudin and Desnues, 2018), owing to its much greater capacity for flexibility and scalability, as well its lower requirement for quantity of input DNA (Valencia et al., 2013).

HCB has an extremely high capacity for multiplexing and scalability of millions of probes, allowing the targeting of extensive genomic regions and very large groups of pathogens. Owing inherently to the relatively long length of probes, HCB enrichment can also achieve high levels of specificity, as high as 98% (Slizovskiy et al., 2022) in targeting appropriate sequences due to lower levels of off-target capture and binding bias, as well as achieving uniformly high coverage of target regions (Pei et al., 2023). Finally, it can achieve very high sensitivity, as high as 99% (Ferreira et al., 2021) and 100% (Slizovskiy et al., 2022), and has been used to sequence viral genomes with 99.99% coverage at >1000x depth from samples with concentrations as low as 2.309 copies/ul (Walker et al., 2024).

The cost per sample of HCB workflows is variable – while certain kits can incur costs as high as $192 USD per sample (Kapel et al., 2023), it can be cost-effective depending on the degree of multiplexing and panel design – VirCapSeq-VERT has been cited as having a per-sample cost of $40 USD in a 20-plex sample workflow (Briese et al., 2015). HCB techniques also require at least an overnight hybridisation as part of the overall workflow (Metsky et al., 2019; Shipley et al., 2020; Zhan et al., 2021). Finally, at least when compared to PCR enrichment, HCB enrichment generally requires a higher level of input DNA.

Panel design is the most critical step in the success of a HCB assay, and several algorithmic tools have been introduced to assist with more efficient and comprehensive capture probe design, such as CATCH (Metsky et al., 2019), HUBDesign (Dickson et al., 2021), and ProbeTools (Kuchinski et al., 2022).

HCB with NGS has been used for a variety of applications on clinical infectious disease samples, including but not limited to pathogen identification, antimicrobial resistance genotyping and whole genome sequencing.

Such an approach greatly improved the sensitivity of virus detection from synthetic metagenomic samples compared with unbiased shotgun metagenomic sequencing (Paskey et al., 2019); for example, Dengue virus was able to be detected down to a strain-specific level at abundance levels as low as ~1000 genome equivalents per ml. Another recently introduced HCB enrichment assay for SARS-CoV-2 detection from nasopharyngeal swab samples achieved a 46,791-fold enrichment of viral reads, with an average of 43% on-target reads and a demonstrated limit of detection of 800 copies/ml (Nagy-Szakal et al., 2021). Indeed, the recent pandemic has spurred much research seeking to apply HCB enrichment to SARS-CoV-2, for purposes including detection but also whole-genome sequencing (Gerber et al., 2022; Meng et al., 2024) and phylogenetic analysis (Orf et al., 2021).

A 13,245-probe Agilent SureSelectXT HCB panel followed by Illumina iSeq100 sequencing was applied directly to human gastric biopsy samples for the characterisation of *Helicobacter pylori* AMR and virulence loci (Gillet et al., 2025). When compared to standard Sanger sequencing as a reference, the HCB-NGS approach achieved 100% and 78.9% concordance at the cagA and vacA loci respectively, while achieving more than 5x coverage at 16S and rpoB resistance loci across the samples tested. The HCB-NGS method could detect *H. pylori* down to a limit of ~1.8×10^5^ CFU/mL (Gillet et al., 2025).

Characterisation of AMR genes from mock human stool samples using HCB enrichment combined with Pacific Biosciences long read sequencing achieved a sensitivity as high as 100% and specificity as high as 98% (Slizovskiy et al., 2022).

Perhaps the most significant application of HCB enrichment as it relates to clinical metagenomics is the introduction of broad-spectrum pathogen enrichment panels which seek to enrich for most, if not all known pathogenic varieties of various microbes. Such an approach seeks to retain the unbiased nature of metagenomics. The most notable example of such a panel is VirCapSeqVERT, a panel comprising approximately 2 million probes which seeks to enrich for all viruses known to infect vertebrates (Briese et al., 2015). Another example of a broad-spectrum viral enrichment panel is the Twist Bioscience Comprehensive Viral Research Panel, a 3153-virus panel which has been notably employed in the recovery of respiratory viruses from rapid antigen tests for whole genome sequencing (Butel-Simoes et al., 2024; Moso et al., 2024).

In cerebrospinal fluid (CSF) samples from suspected viral meningitis patients, VirCapSeqVERT was able to detect a clinically relevant virus in 9 out of 16 cases (McGill et al., 2022). When applied to nasopharyngeal swab samples from clusters of previously unexplained Severe Acute Respiratory Infection cases, the method could detect respiratory viruses in 81.8% of samples, while detecting coinfection with another virus in 50.7% of samples (Cummings et al., 2019). Finally, another study sought to apply both VirCapSeqVERT and BacCapSeq, a similar panel which seeks to comprehensively enrich for pathogenic bacteria, to CSF samples from 34 patients with either definitive or presumed CNS infections (Boruah et al., 2023). The expected pathogen could be confirmed in 42.8% of cases with definite infection, while pathogens could be detected in 42% of the suspected cases, however often these were commensal microbes.

### 3.6 Syndromic panels for specific infections

An emerging enrichment approach in the field of NGS diagnosis for infectious diseases is the use of syndromic panels, which seek to enrich specifically for pathogens associated with specific clinical syndromes including respiratory infections, central nervous system (CNS) infections, bloodstream infections, and urinary tract infections. Syndromic panels tested to date have employed either multiplex PCR-based or hybrid capture-based enrichment (Li et al., 2022; Almas et al., 2023; Chen et al., 2024; Wang et al., 2024; Yin et al., 2024).

Such panels may represent a useful clinical tool in the workup of patients with these specific presentations, especially in cases such as CNS infections where a causative pathogen, if any, is often difficult to identify and where an unbiased metagenomic approach sometimes carries an excessive bioinformatic burden and prohibitive sequencing costs (Chen et al., 2024). Furthermore, syndromic panels can often integrate characterisation of antimicrobial resistance gene detection, supporting the timely optimisation of antimicrobial therapy and potentially leading to better patient outcomes (Li et al., 2022; Wang et al., 2024). Finally, using an approach that targets common causative microbes for a specific infective syndrome increases the confidence that any microorganisms detected are in fact the causative pathogen in that case, rather than simply being part of the normal flora or an unintentional contaminant (Ramanan et al., 2018).

The main downside to syndromic panels, as is true to an extent for most targeted NGS strategies, is the fact that the utility of the panels is contingent on comprehensive and up-to-date pathogen coverage for the targeted syndrome, with panels needing to be meticulously designed to ensure diagnostic accuracy and relevance, accounting for genetic diversity and evolving resistance mechanisms (Chen et al., 2024; Yin et al., 2024). Such panels are therefore limited in their detection of unexpected or novel pathogens. Furthermore, the fact that panel design requires subjective input risks introducing bias in terms of the organisms selected, the coverage of each organism in the panel, and the design of the probes or primers themselves, potentially leading to misleading results (McGill et al., 2022; Boruah et al., 2023; Pei et al., 2023). Nevertheless, such panels have already shown promise in translating enrichment-based approaches into improving detection of pathogens in clinical infections.

A >300-plex PCR panel targeting 198 common respiratory pathogens, alongside an ultra-high multiplex HCB probe panel targeting >3000 common and rare respiratory pathogens, both followed by Illumina MiniSeq sequencing, were employed for the diagnosis of respiratory tract infections in bronchoalveolar lavage fluid samples (Yin et al., 2024). The study found sensitivities of 86.5% (PCR) and 87.3% (HCB) compared to 85.5% for mNGS, and specificities of 90.0% (PCR) and 88.0% (HCB) compared to 92.1% for mNGS, in the identification of pathogens in the samples (Yin et al., 2024). This was despite a significantly lower cost per sample for the syndromic panels compared to a metagenomic approach, at ~120$ for PCR and ~300$ for HCB compared to ~500-900$ for mNGS (Yin et al., 2024). The limits of detection for the two panels ranged from 50–450 CFU/ml (Yin et al., 2024).A 107-pathogen HCB panel (followed by KM MiniSeqDx-CN sequencing) targeting common respiratory tract pathogens was applied for the diagnosis of paediatric respiratory tract infections from throat swab samples, achieving a sensitivity of 91.38% compared to 29.68% for conventional microbiological testing applied to the same samples (Wang et al., 2024). Furthermore, in several cases, the targeted syndromic panel was able to identify mixed infections and low abundance RNA viruses which were not detected by the conventional tests (Wang et al., 2024). Another 306-organism HCB panel targeting respiratory pathogens was applied to respiratory swab, bronchoalveolar lavage fluid, and sputum samples from patients with suspected lower respiratory tract infections, followed by Gene+ Seq-100 sequencing, for pathogen identification and AMR profiling (Gu et al., 2025). The assay achieved a sensitivity of 97.73% and specificity of 75.41%, while reaching an accuracy of 80.56% in identification of AMR markers when compared with antimicrobial susceptibility tests (Gu et al., 2025).

524-primer PCR syndromic panel (followed by Illumina MiSeq sequencing) targeting the leading reported causative pathogens for CNS infections was applied to CSF samples for the diagnosis of meningitis and encephalitis cases, and compared to an equivalent mNGS approach (Chen et al., 2024). The syndromic panel approach achieved significantly higher diagnostic accuracy, positive percent agreement and negative percent agreement than mNGS in identifying the causative pathogens in the samples, at 65.1%, 64.0% and 66.7% compared to 47.4%, 43.8% and 52.4%, respectively (Chen et al., 2024).

A 920-plex Ampliseq PCR panel targeting 47 common bloodstream pathogens for identification and antimicrobial resistance profiling, followed by Ion Torrent S5 sequencing, was applied to several clinical blood samples from patients with potential bloodstream infections in order to validate the technique (Li et al., 2022). Of the 5 samples which had tested positive on gold standard blood culture, the Ampliseq panel successfully identified the causative pathogen in 4 of them, while the 3 negative blood culture samples were also found to be negative via the Ampliseq approach (Li et al., 2022). For the 1 sample which was misidentified by the Ampliseq panel, containing *Staphylococcus hominis*, it was found that there was an insufficient number of *S. hominis*-specific primers in the panel, highlighting the potential limitations still inherent to targeted approaches such as this (Li et al., 2022). Overall, it was concluded that further testing of the technique on real blood samples was needed to validate its clinical utility (Li et al., 2022).

A HCB panel targeting 191 causative pathogens of urinary tract infections (UTI’s), followed by Illumina MiniSeq sequencing, was applied to urine samples from patients with clinically suspected UTI’s to investigate the diagnostic yield of this approach as compared to traditional culture and non-NGS PCR-based methods (Almas et al., 2023). The syndromic panel approach notably increased the number of different organisms detected among the samples compared to culture and PCR-based assays, with 62 different microbes identified among the samples compared with 13 and 19 for culture and PCR respectively (Almas et al., 2023). Furthermore, the HCB-NGS approach showed superior performance in detecting polymicrobial infections, a common occurrence in UTI’s, identifying them in 98% of samples compared with 83% for PCR and 70% for culture (Almas et al., 2023).

## 4 Conclusion

Various enrichment techniques are currently available for the augmentation of mNGS, each with their own unique benefits and downsides. PCR-based enrichment is cost-effective and time-efficient; CRISPR FLASH benefits from flexibility and high sensitivity; MIPs offer the ability for highly multiplexed assays at low cost; AS provides the unique benefits of real-time enrichment and analysis without extensive sample preparation; HCB enrichment can offer unparalleled large multiplexed panels with high specificity, although with the caveat of higher cost. Overall, these enrichment approaches can augment the use of mNGS, particularly in situations where pathogen load is very low – albeit with the crucial consideration that this negates the hypothesis-free nature of shotgun mNGS, These techniques have been utilised for various purposes such as pathogen detection, AMR profiling, and whole-genome sequencing from a diverse range of clinical sample types. Nevertheless, more research is needed to further refine the application of these enrichment techniques to future, real-world metagenomic diagnostics.
